# Supplementary material for: Shifts in pore connectivity from precipitation versus groundwater rewetting increases soil carbon loss after drought
Source: Nat Commun. 2017 Nov 6;8:1335. doi: 10.1038/s41467-017-01320-x (PMC5673896; doi:10.1038/s41467-017-01320-x)
Supplement: Supplementary file 1 — Supplementary Information [file 41467_2017_1320_MOESM1_ESM.pdf]

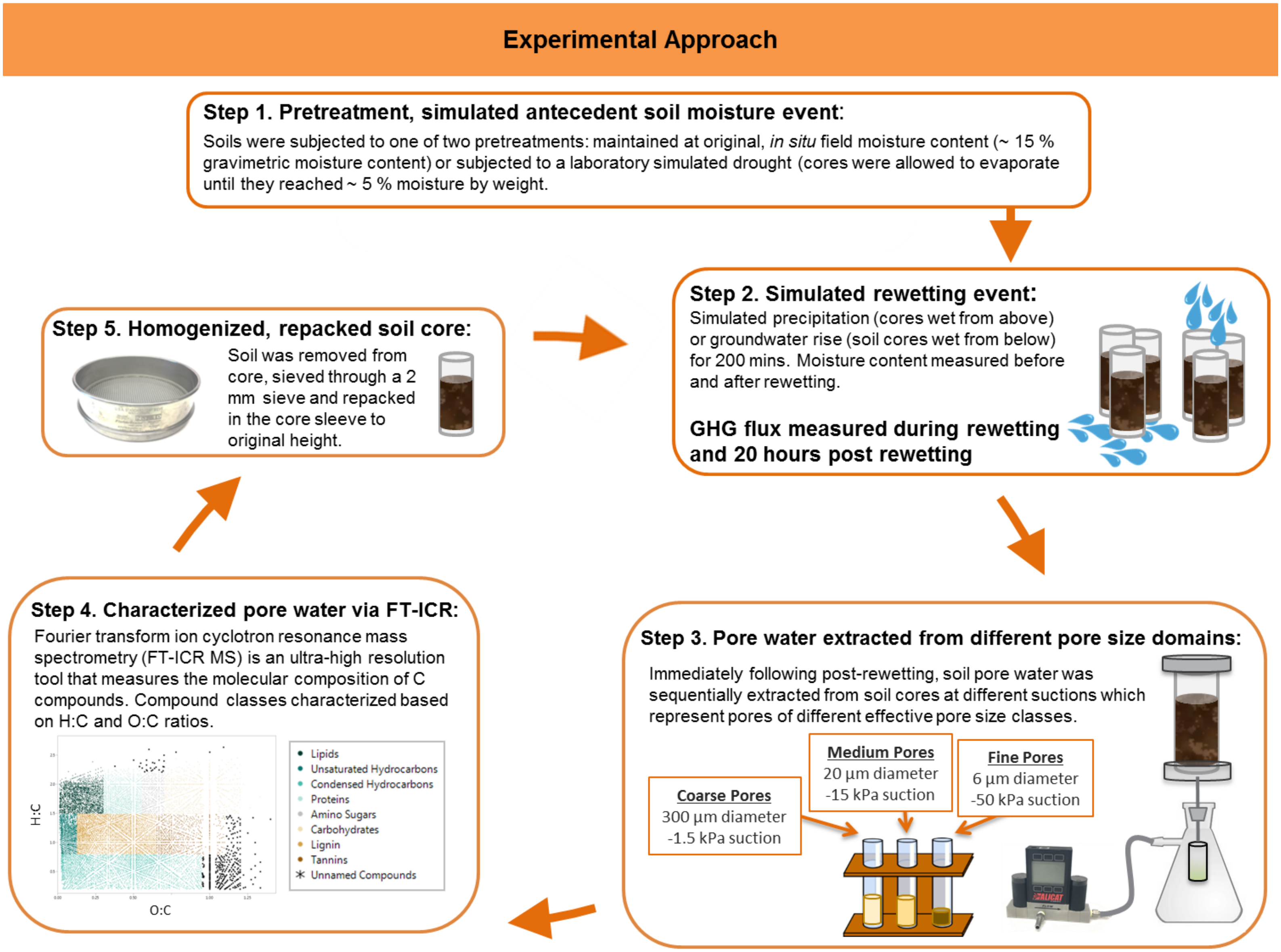

Supplementary Figure 1. Experimental Approach. Diagram outlining the approach used to test the combined effects of antecedent drought, wetting direction, and soil homogenization on core- and pore-scale C transformations, including (1) soil pre-treatment to establish antecedent moisture conditions, (2) soil rewetting and incubation and carbonaceous greenhouse gas (GHG) measurements, (3) collection and (4) characterization of pore water from different effective pore size domains, (5) soil homogenization and core repacking, and rerunning experiment (Steps 2 - 4) on repacked, homogenized soil cores.

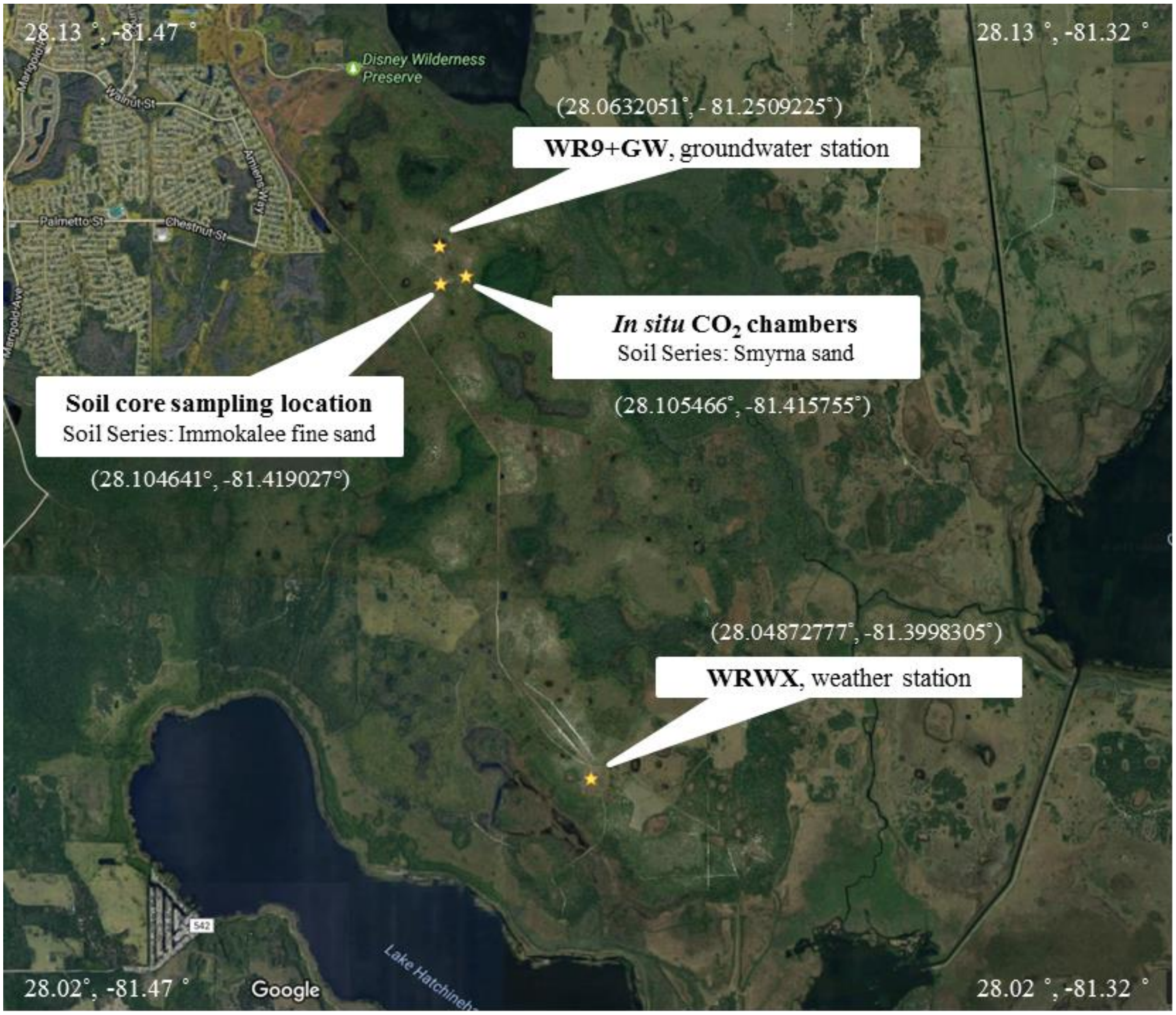

Map data ©2016 Google

Supplementary Figure 2. Map of field sites at the Disney Wilderness Preserve, FL. Google map showing the locations (latitude, longitude) used in our study; collection site for intact soil cores, field sites for *in situ* CO_2 measurements (CO_2 chambers), groundwater station (WR9+GW1), and weather tower (WRWX). Total distance covered represents 12 by 15 kilometers.

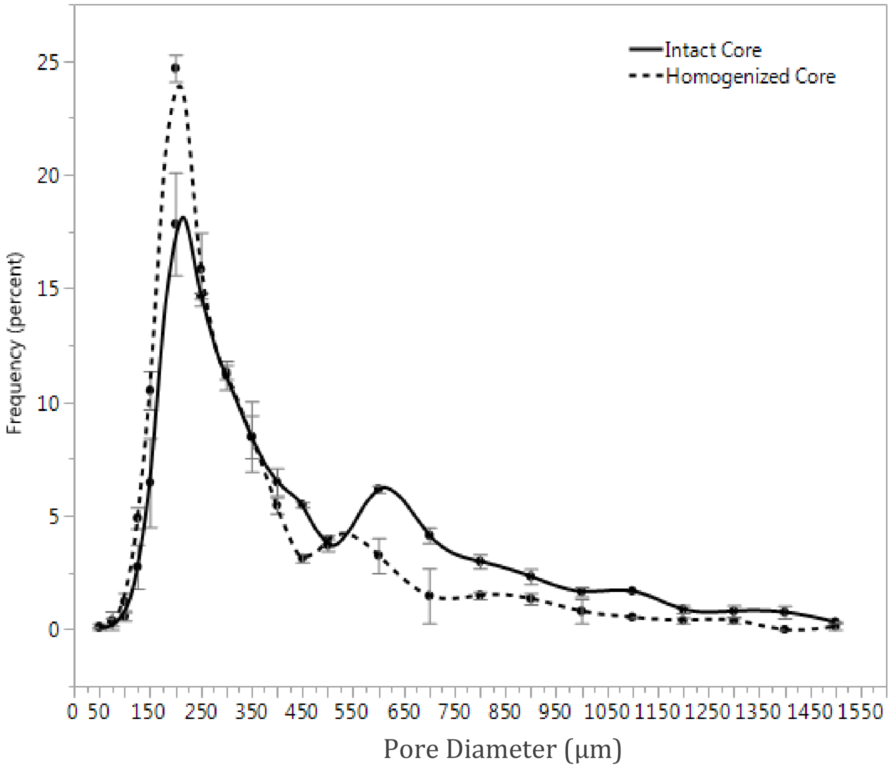

Supplementary Figure 3. Pore size distribution as determined by X-ray computed tomography for an intact and homogenized soil core. Soil cores were scanned using X-ray Computed Tomography (XCT) on an X-Tek/Metris XTH 320/225 kV scanner (Nikon Metrology, Belmont, CA). Data was collected at 110 kV and 265 µA X-ray power. The core samples were rotated continuously during the scans with momentary stops to collect each projection (shuttling mode) while minimizing ring artifacts. A total of 3142 projections were collected over 360˚ with 0.5 second exposure time and 4 frames per projection. Image voxel size was 28 microns. The images were reconstructed to obtain three-dimensional datasets using CT Pro 3D (Metris XT 2.2, Nikon Metrology). Representative slice and 3D images were created using VG Studio MAX 2.1 (Volume Graphics GmbH, Heidelberg Germany). Image processing and porosity analysis (including pore volume segmentation and pore analysis) was carried out using ImageJ 1.51k (National Institute of Health, USA). Two subsections of the scans were used for each core to create the pore size distributions shown. Bars represent standard error.

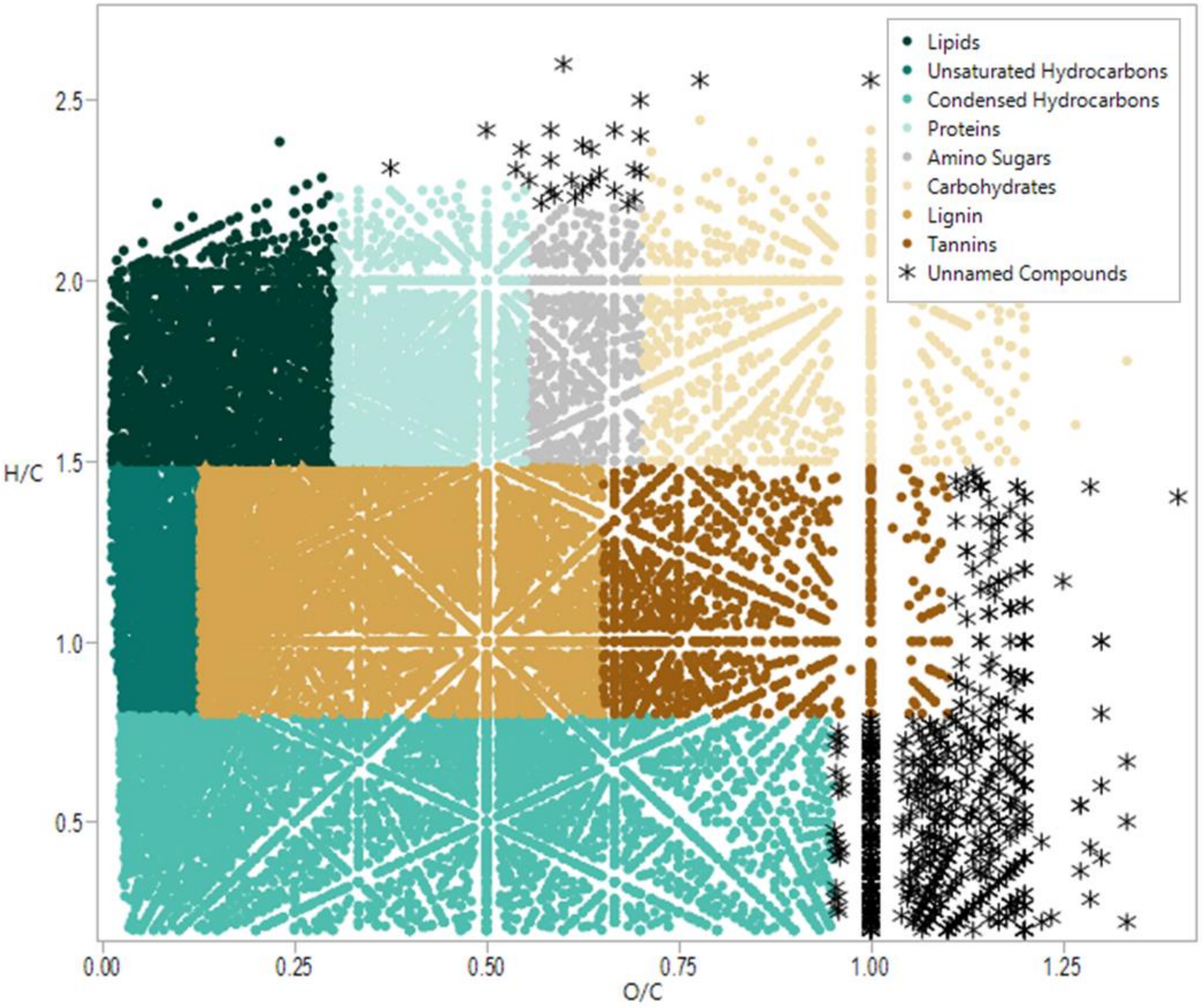

Supplementary Figure 4. Molecular compound classifications plotted on a van Krevelen diagram. The ranges of H/C and O/C for each molecular compound class used in our analysis of the composition of soil pore water plotted on a van Krevelen diagram. FT-ICR spectra were classified into eight biomolecular groups, referred to as FT-ICR compound classes, based on O/C and H/C counts; lipids ($0 < O/C \leq 0.3$, $1.5 \leq H/C \leq 2.5$), unsaturated hydrocarbons ($0 \leq O/C \leq 0.125$, $0.8 \leq H/C < 2.5$), proteins ($0.3 < O/C \leq 0.55$, $1.5 \leq H/C \leq 2.3$), amino sugars ($0.55 < O/C \leq 0.7$, $1.5 \leq H/C \leq 2.2$), carbohydrates ($0.7 < O/C \leq 1.5$, $1.5 \leq H/C \leq 2.5$), lignin ($0.125 < O/C \leq 0.65$, $0.8 \leq H/C < 1.5$), tannins ($0.65 < O/C \leq 1.1$, $0.8 \leq H/C < 1.5$), and condensed hydrocarbons ($0 \leq O/C \leq 0.95$, $0.2 \leq H/C < 0.8$).

	Correlation Coefficients										
	PCA axis 1	PCA axis 2	Unnamed Compounds	Condensed Hydrocarbons	Tannins	Lignin	Carbohydrates	Amino Sugars	Proteins	Unsaturated Hydrocarbons	Lipids
**Unnamed Compounds**	-96.7%	0.0%	100.0%	-51.4%	-59.5%	-86.0%	-19.5%	-55.4%	-49.5%	-43.9%	18.6%
**Condensed Hydrocarbons**	54.1%	-67.3%	-51.4%	100.0%	74.6%	31.5%	-22.7%	-11.0%	-27.2%	22.9%	-61.3%
**Tannins**	64.4%	-63.9%	-59.5%	74.6%	100.0%	51.9%	-8.1%	0.6%	-25.4%	14.3%	-65.7%
**Lignin**	75.8%	-14.5%	-86.0%	31.5%	51.9%	100.0%	4.9%	27.1%	27.8%	6.9%	-26.1%
**Carbohydrates**	25.5%	59.5%	-19.5%	-22.7%	-8.1%	4.9%	100.0%	53.4%	41.8%	18.2%	35.3%
**Amino Sugars**	63.5%	68.5%	-55.4%	-11.0%	0.6%	27.1%	53.4%	100.0%	86.2%	56.9%	29.1%
**Proteins**	48.6%	80.9%	-49.5%	-27.2%	-25.4%	27.8%	41.8%	86.2%	100.0%	44.0%	52.5%
**UnSaturated Hydrocarbons**	57.7%	24.1%	-43.9%	22.9%	14.3%	6.9%	18.2%	56.9%	44.0%	100.0%	-12.3%
**Lipids**	-28.8%	80.9%	18.6%	-61.3%	-65.7%	-26.1%	35.3%	29.1%	52.5%	-12.3%	100.0%

Correlation ≥ 90 %
Correlation ≥ 80 %
Correlation ≥ 70 %
Correlation ≥ 60 %
Correlation ≥ 50 %

Supplementary Table 1. Correlation coefficients of FT-ICR compound classes with Principal Component axes and other compound classes. The correlation coefficients of principal component analysis (PCA) factors (shown in **Figure 2**) with principal component axes and between other factors in the PCA are shown. Factors included in the PCA were relative abundance values of Fourier-transform ion cyclotron resonance (FT-ICR) mass spectrometry defined organic carbon compound classes (lipid, unsaturated hydrocarbons, lignin, proteins, etc.) for soil pore water collected at -1.5, -15 and -50 kPa suctions from intact and repack, homogenized cores subjected to different moisture content and wetting direction treatments. Darker blue shading indicates greater correlation (>50%).

Core Structure	Pore Water Fraction	Moisture	Wetting Direction	N	Lipids	Unsaturated Hydrocarbons	Condensed Hydrocarbons	Proteins	Amino Sugars	Carbohydrates	Lignin	Tannins	Unnamed
**Intact**	-1.5 kPa	Field Moisture	precipitation	4	0.066 ± 0.015	0.029 ± 0.011	0.078 ± 0.018	0.066 ± 0.012	0.012 ± 0.003	0.006 ± 0.002	0.142 ± 0.012	0.012 ± 0.004	0.588 ± 0.026
			groundwater rise	4	0.081 ± 0.011	0.018 ± 0.006	0.094 ± 0.007	0.065 ± 0.014	0.015 ± 0.003	0.009 ± 0.001	0.145 ± 0.035	0.021 ± 0.004	0.554 ± 0.044
		Antecedent Drought	precipitation	4	0.051 ± 0.005	0.016 ± 0.006	0.088 ± 0.006	0.057 ± 0.009	0.013 ± 0.002	0.008 ± 0.001	0.196 ± 0.051	0.019 ± 0.004	0.551 ± 0.073
			groundwater rise	4	0.051 ± 0.007	0.025 ± 0.008	0.101 ± 0.011	0.043 ± 0.006	0.011 ± 0.002	0.009 ± 0.002	0.107 ± 0.016	0.015 ± 0.004	0.638 ± 0.034
	-15 kPa	Field Moisture	precipitation	4	0.055 ± 0.009	0.026 ± 0.010	0.099 ± 0.018	0.061 ± 0.009	0.012 ± 0.001	0.005 ± 0.001	0.265 ± 0.050	0.024 ± 0.006	0.453 ± 0.060
			groundwater rise	4	0.058 ± 0.008	0.041 ± 0.006	0.090 ± 0.016	0.077 ± 0.020	0.019 ± 0.006	0.010 ± 0.002	0.186 ± 0.047	0.023 ± 0.010	0.496 ± 0.093
		Antecedent Drought	precipitation	3	0.048 ± 0.012	0.029 ± 0.014	0.103 ± 0.020	0.071 ± 0.017	0.019 ± 0.005	0.010 ± 0.002	0.263 ± 0.084	0.037 ± 0.017	0.421 ± 0.111
			groundwater rise	3	0.060 ± 0.005	0.068 ± 0.029	0.098 ± 0.003	0.115 ± 0.015	0.027 ± 0.006	0.016 ± 0.003	0.272 ± 0.022	0.025 ± 0.002	0.319 ± 0.034
	-50 kPa	Field Moisture	precipitation	4	0.047 ± 0.008	0.028 ± 0.008	0.129 ± 0.016	0.067 ± 0.011	0.015 ± 0.002	0.007 ± 0.002	0.227 ± 0.019	0.030 ± 0.011	0.450 ± 0.019
			groundwater rise	3	0.046 ± 0.008	0.041 ± 0.016	0.090 ± 0.026	0.072 ± 0.003	0.016 ± 0.003	0.007 ± 0.001	0.228 ± 0.015	0.026 ± 0.008	0.475 ± 0.027
		Antecedent Drought	precipitation	4	0.038 ± 0.004	0.041 ± 0.011	0.127 ± 0.019	0.071 ± 0.011	0.014 ± 0.002	0.008 ± 0.002	0.316 ± 0.013	0.042 ± 0.009	0.342 ± 0.027
			groundwater rise	2	0.049 ± 0.016	0.026 ± 0.020	0.112 ± 0.014	0.063 ± 0.040	0.010 ± 0.007	0.008 ± 0.000	0.211 ± 0.011	0.016 ± 0.002	0.506 ± 0.055
**Homogenized**	-1.5 kPa	Field Moisture	precipitation	4	0.066 ± 0.008	0.039 ± 0.011	0.104 ± 0.011	0.084 ± 0.021	0.018 ± 0.003	0.008 ± 0.001	0.206 ± 0.038	0.014 ± 0.006	0.461 ± 0.037
			groundwater rise	4	0.051 ± 0.006	0.033 ± 0.008	0.095 ± 0.005	0.054 ± 0.011	0.012 ± 0.003	0.007 ± 0.001	0.154 ± 0.014	0.016 ± 0.004	0.577 ± 0.033
		Antecedent Drought	precipitation	4	0.046 ± 0.007	0.022 ± 0.009	0.110 ± 0.020	0.055 ± 0.011	0.012 ± 0.004	0.008 ± 0.002	0.226 ± 0.047	0.027 ± 0.012	0.495 ± 0.063
			groundwater rise	3	0.029 ± 0.001	0.041 ± 0.001	0.173 ± 0.014	0.029 ± 0.005	0.007 ± 0.001	0.005 ± 0.000	0.21 ± 0.012	0.043 ± 0.005	0.464 ± 0.019
	-15 kPa	Field Moisture	precipitation	4	0.068 ± 0.011	0.034 ± 0.008	0.092 ± 0.005	0.084 ± 0.016	0.014 ± 0.004	0.007 ± 0.001	0.220 ± 0.018	0.010 ± 0.001	0.471 ± 0.047
			groundwater rise	4	0.045 ± 0.009	0.028 ± 0.010	0.125 ± 0.028	0.050 ± 0.012	0.009 ± 0.003	0.006 ± 0.001	0.227 ± 0.014	0.032 ± 0.017	0.479 ± 0.059
		Antecedent Drought	precipitation	3	0.022 ± 0.003	0.038 ± 0.004	0.190 ± 0.036	0.042 ± 0.004	0.010 ± 0.002	0.005 ± 0.001	0.275 ± 0.028	0.068 ± 0.017	0.350 ± 0.081
			groundwater rise	3	0.060 ± 0.016	0.065 ± 0.015	0.101 ± 0.019	0.086 ± 0.027	0.024 ± 0.009	0.019 ± 0.009	0.204 ± 0.063	0.030 ± 0.015	0.410 ± 0.122
	-50 kPa	Field Moisture	precipitation	3	0.063 ± 0.015	0.052 ± 0.006	0.135 ± 0.012	0.089 ± 0.010	0.017 ± 0.001	0.007 ± 0.001	0.225 ± 0.008	0.014 ± 0.005	0.398 ± 0.008
			groundwater rise	4	0.036 ± 0.006	0.040 ± 0.008	0.152 ± 0.029	0.056 ± 0.012	0.014 ± 0.003	0.007 ± 0.001	0.226 ± 0.025	0.046 ± 0.012	0.424 ± 0.048
		Antecedent Drought	precipitation	3	0.038 ± 0.011	0.018 ± 0.007	0.143 ± 0.041	0.060 ± 0.010	0.014 ± 0.001	0.013 ± 0.004	0.317 ± 0.069	0.052 ± 0.025	0.346 ± 0.118
			groundwater rise	3	0.042 ± 0.012	0.065 ± 0.020	0.127 ± 0.031	0.075 ± 0.026	0.018 ± 0.007	0.010 ± 0.003	0.264 ± 0.021	0.046 ± 0.015	0.355 ± 0.019

Supplementary Table 2. Relative abundance of FT-ICR compound classes across antecedent drought, wetting direction and soil homogenization treatments for each effective pore size domain. The mean and standard error for each FT-ICR compound classes used in our analysis for all treatment combinations and for each pore water fraction (-1.5 kPa, -15 kPa, and -50 kPa) as presented in Figure 2. The number of replicates for each group is reported as N.

Core Structure	Pore Water Fraction	Moisture	Wetting Direction	N	**WSOC** (mg L^{-1})	**WSN** (mg L^{-1})	**pore water volume** (ml)
Intact	-1.5 kPa	Field Moisture	precipitation	4	6.43 ± 1.20	0.00 ± 0.00	12.45 ± 1.14
			groundwater rise	4	10.47 ± 1.98	0.00 ± 0.00	14.47 ± 3.77
		Antecedent Drought	precipitation	4	8.17 ± 3.70	0.91 ± 0.91	14.54 ± 1.96
			groundwater rise	4	1.50 ± 1.50	0.00 ± 0.00	15.84 ± 3.50
	-15 kPa	Field Moisture	precipitation	4	14.05 ± 2.48	0.00 ± 0.00	2.20 ± 0.37
			groundwater rise	3	24.01 ± 6.08	1.28 ± 0.64	1.92 ± 0.29
		Antecedent Drought	precipitation	3	33.01 ± 3.10	2.75 ± 0.54	2.33 ± 1.67
			groundwater rise	2	36.65 ± 21.11	3.17 ± 0.70	2.84 ± 1.04
	-50 kPa	Field Moisture	precipitation	3	16.08 ± 1.22	0.00 ± 0.00	5.32 ± 0.92
			groundwater rise	3	23.80 ± 4.42	0.62 ± 0.62	3.68 ± 1.10
		Antecedent Drought	precipitation	4	35.88 ± 2.59	3.39 ± 1.38	4.30 ± 1.20
			groundwater rise	2	23.46 ± 4.21	1.28 ± 1.28	6.99 ± 0.94
Homogenized	-1.5 kPa	Field Moisture	precipitation	4	7.70 ± 2.31	0.00 ± 0.00	16.74 ± 0.99
			groundwater rise	4	12.88 ± 1.53	0.56 ± 0.56	16.23 ± 2.36
		Antecedent Drought	precipitation	4	844.88 ± 480.20	0.74 ± 0.74	14.73 ± 2.74
			groundwater rise	3	267.60 ± 255.46	1.63 ± 1.63	23.16 ± 2.85
	-15 kPa	Field Moisture	precipitation	4	16.91 ± 1.22	0.00 ± 0.00	3.66 ± 0.67
			groundwater rise	4	23.10 ± 2.62	1.52 ± 0.88	2.50 ± 0.56
		Antecedent Drought	precipitation	2	25980.50 ± 24699.50	4.82 ± 2.43	3.70 ± 1.97
			groundwater rise	3	5872.27 ± 5760.17	1.00 ± 1.00	2.09 ± 0.82
	-50 kPa	Field Moisture	precipitation	3	16.26 ± 1.12	0.59 ± 0.59	3.52 ± 0.20
			groundwater rise	4	22.88 ± 3.26	0.99 ± 0.58	4.10 ± 1.04
		Antecedent Drought	precipitation	3	18001.97 ± 9462.00	5.57 ± 3.42	2.39 ± 0.20
			groundwater rise	2	17512.40 ± 10519.61	1.16 ± 1.16	1.32 ± 0.88

Supplementary Table 3. Water soluble organic carbon and nitrogen in pore water from intact and homogenized cores across treatment combinations of antecedent drought, and rewetting direction. Mean and standard error values for water soluble organic carbon (WSOC, mg L^{-1}) and nitrogen (WSN, mg L^{-1}), and pore water volumes in pore water collected from all treatment combinations, separated by effective pore size domains (pore water fractions; -1.5, -15 and -50 kPa suctions). Replication is included under column "N". Pore water was collected from individual soil cores immediately following wetting and post-wetting incubation.

Core Structure	Effect	WSOC (mg L^{-1})	WSN (mg L^{-1})	pore water volume (ml)
**Intact**	Pore Water Fraction	**<.0001**	**0.0005**	**<.0001**
	Antcedent Drought	ns	**0.0056**	ns
	Rewetting Direction	ns	ns	ns
	Pore Water Fraction*Antcedent Drought	**0.0028**	**0.0414**	ns
	Pore Water Fraction*Rewetting Direction	ns	ns	ns
	Antcedent Drought*Rewetting Direction	**0.0144**	ns	ns
	Pore Water Fraction*Antcedent Drought*Rewetting Dir	ns	ns	ns
**Homogenized**	Pore Water Fraction	**<.0001**	ns	**<.0001**
	Antcedent Drought	**0.0006**	ns	ns
	Rewetting Direction	ns	ns	ns
	Pore Water Fraction*Antcedent Drought	**0.0022**	ns	ns
	Pore Water Fraction*Rewetting Direction	ns	ns	ns
	Antcedent Drought*Rewetting Direction	ns	ns	ns
	Pore Water Fraction*Antcedent Drought*Rewetting Dir	ns	ns	ns

p-values in **bold** are significant at $p < 0.05$

Supplementary Table 4. Statistical summary of treatment effect on water soluble organic carbon and nitrogen in pore waters from intact and homogenized cores. P-values from residual maximum likelihood models (REML) performed on log transformed data, with an added integer of 1 to avoid errors from calculating the log of zero. P-values are included when effect is significant ($p < 0.05$), whereas non-significant effects are denoted by ns.

Core Scale Properties		Intact				Homogenized			
		Field Moisture		Antecedent Drought		Field Moisture		Antecedent Drought	
		precipitation	groundwater	precipitation	groundwater	precipitation	groundwater	precipitation	groundwater
**Soil Properties**									
N		4	4	4	4	4	4	4	3
Bulk Density (ρ_b)		1.15 ± 0.02	1.06 ± 0.11	1.19 ± 0.03	1.17 ± 0.02	1.13 ± 0.02	1.02 ± 0.11	1.25 ± 0.02	1.24 ± 0.02
Est. Porosity (φ)		0.57 ± 0.01	0.60 ± 0.04	0.55 ± 0.01	0.56 ± 0.01	0.57 ± 0.01	0.62 ± 0.04	0.53 ± 0.01	0.53 ± 0.01
Soil N (g/ 100g soil)		0.04 ± 0.00	0.04 ± 0.01	0.03 ± 0.00	0.03 ± 0.00	0.02 ± 0.00	0.03 ± 0.01	0.02 ± 0.00	0.03 ± 0.00
Soil C (g/ 100g soil)		0.69 ± 0.17	0.89 ± 0.23	0.48 ± 0.03	0.43 ± 0.04	0.36 ± 0.03	0.60 ± 0.26	0.46 ± 0.03	0.47 ± 0.05
Molar C:N		21.71 ± 2.36	24.08 ± 3.15	21.55 ± 1.12	19.47 ± 0.54	20.01 ± 0.68	24.16 ± 4.68	23.34 ± 1.11	21.64 ± 0.53
**Moisture Properties**									
Pre-Wetting Moisture Content									
	gravimetric	0.12 ± 0.03	0.14 ± 0.04	0.06 ± 0.02	0.03 ± 0.01	0.08 ± 0.04	0.05 ± 0.02	0.05 ± 0.02	0.05 ± 0.01
	volumetric	0.14 ± 0.03	0.14 ± 0.04	0.07 ± 0.02	0.04 ± 0.01	0.09 ± 0.05	0.05 ± 0.02	0.07 ± 0.02	0.06 ± 0.01
Post-Wetting Moisture Content									
	gravimetric	0.24 ± 0.01	0.25 ± 0.02	0.26 ± 0.02	0.23 ± 0.02	0.28 ± 0.06	0.24 ± 0.03	0.26 ± 0.02	0.33 ± 0.03
	volumetric	0.27 ± 0.01	0.26 ± 0.01	0.30 ± 0.02	0.27 ± 0.02	0.32 ± 0.07	0.24 ± 0.02	0.33 ± 0.03	0.41 ± 0.03
Water Imbibed (ml)		13.08 ± 4.18	13.55 ± 3.77	27.14 ± 0.32	26.25 ± 1.95	25.38 ± 5.09	22.6 ± 3.35	28.97 ± 0.23	36.75 ± 3.06
Gas-filled pore space displaced by water (%)		22.91 ± 7.2	23.07 ± 6.78	49.15 ± 1.19	47.08 ± 3.85	44.48 ± 9.35	38.07 ± 7.27	54.82 ± 1.03	69.13 ± 6.44

Supplementary Table 5. Core-scale physical, chemical and moisture properties. Mean and standard error values for select soil and moisture properties for intact and repacked, homogenized cores either maintained at field moisture content or subjected to laboratory-induced antecedent drought before and after rewetting from above (simulated precipitation) or from below (simulated groundwater rise). Soil properties were measured after pore water was collected from each soil core (i.e. when the experiment was concluded), whereas moisture properties were measured before and after rewetting treatment.

Statisitcal Significance Summary	P-values Soil N	Soil C	Molar C:N	Pre-Wetting Moisture Content gravimetric	Pre-Wetting Moisture Content volumetric	Post-Wetting Moisture Content gravimetric	Post-Wetting Moisture Content volumetric	Water Imbibed	Gas-filled pore space displaced by water
**Intact Soil Cores**									
Antecedent drought	$p = 0.017$	$p = 0.038$	ns	$p = 0.009$	$p = 0.012$	ns	ns	$p = 0.001$	$p = 0.001$
Wetting Direction	ns	ns	ns	ns	ns	ns	ns	ns	ns
Antecedent Drought * Wetting Direction	ns	ns	ns	ns	ns	ns	ns	ns	ns
**Homgenized Soil Cores**									
Antecedent drought	ns	ns	ns	ns	ns	ns	ns	$p = 0.028$	$p = 0.012$
Wetting Direction	ns	ns	ns	ns	ns	ns	ns	ns	ns
Antecedent Drought * Wetting Direction	ns	ns	ns	ns	ns	ns	ns	ns	ns

ns = no significant effect

Supplementary Table 6. Statistical significance for treatment effects (antecedent drought and wetting direction) on core-scale chemical and moisture properties. P-values for main and interactive effects; antecedent drought and wetting direction, for soil chemical properties (C, N, etc.) and moisture properties displayed in **Supplementary Table 4**.
