# Supplementary material for: Shifts in pore connectivity from precipitation versus groundwater rewetting increases soil carbon loss after drought
Source: Nat Commun. 2017 Nov 6;8:1335. doi: 10.1038/s41467-017-01320-x (PMC5673896; doi:10.1038/s41467-017-01320-x)
Supplement: Supplementary file 4 — Supplementary Movie 1 [file 41467_2017_1320_MOESM4_ESM.pptx]

## Slide 1
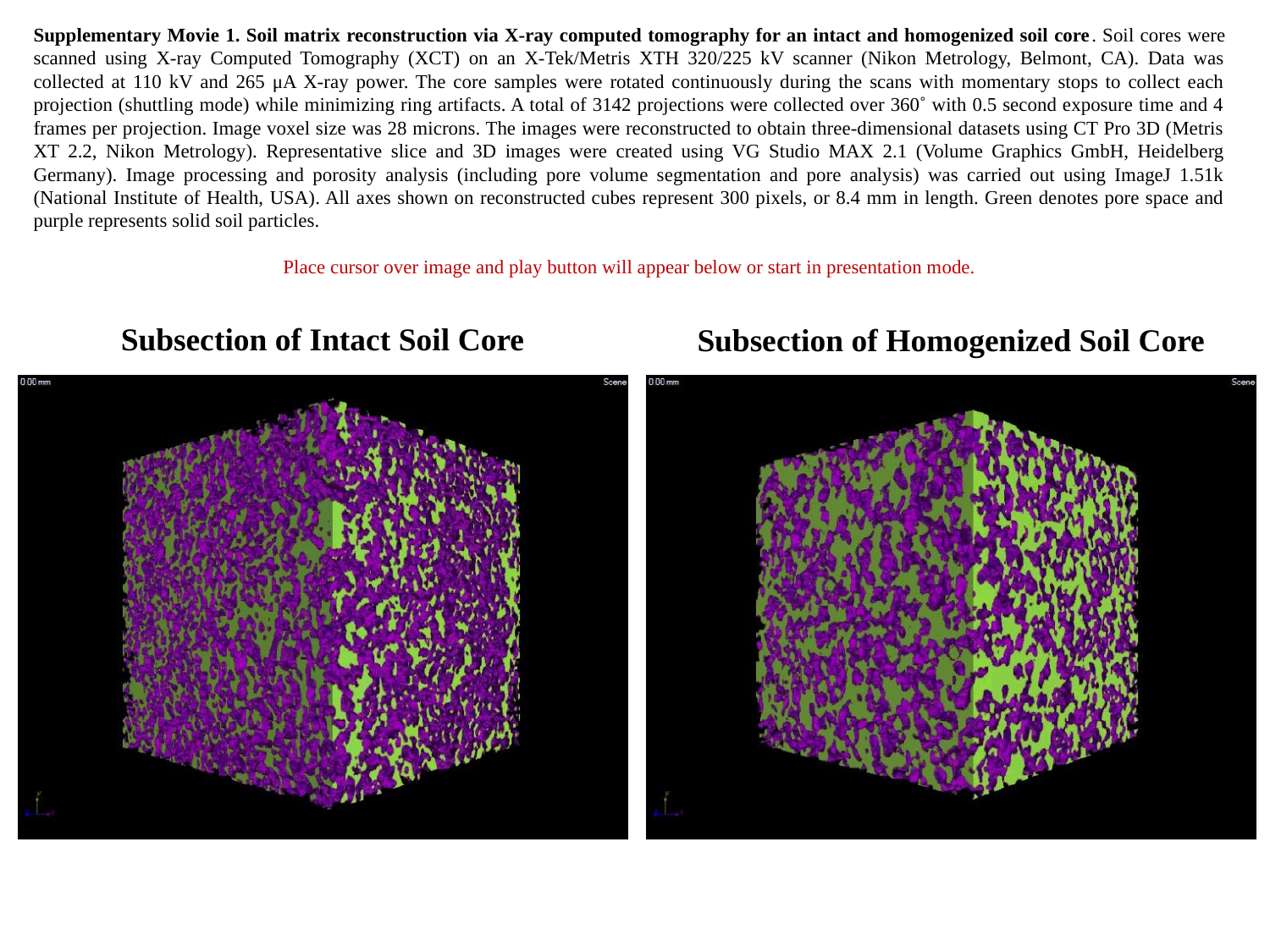

Supplementary Movie 1. Soil matrix reconstruction via X-ray computed tomography for an intact and homogenized soil core. Soil cores were scanned using X-ray Computed Tomography (XCT) on an X-Tek/Metris XTH 320/225 kV scanner (Nikon Metrology, Belmont, CA). Data was collected at 110 kV and 265 μA X-ray power. The core samples were rotated continuously during the scans with momentary stops to collect each projection (shuttling mode) while minimizing ring artifacts. A total of 3142 projections were collected over 360˚ with 0.5 second exposure time and 4 frames per projection. Image voxel size was 28 microns. The images were reconstructed to obtain three-dimensional datasets using CT Pro 3D (Metris XT 2.2, Nikon Metrology). Representative slice and 3D images were created using VG Studio MAX 2.1 (Volume Graphics GmbH, Heidelberg Germany). Image processing and porosity analysis (including pore volume segmentation and pore analysis) was carried out using ImageJ 1.51k (National Institute of Health, USA). All axes shown on reconstructed cubes represent 300 pixels, or 8.4 mm in length. Green denotes pore space and purple represents solid soil particles.
Place cursor over image and play button will appear below or start in presentation mode.
Subsection of Intact Soil Core
Subsection of Homogenized Soil Core
